# Low-frequency EEG correlates of fMRI in the resting state

**DOI:** 10.1186/1471-2202-13-S1-P107

**Published:** 2012-07-16

**Authors:** Joshua K Grooms, Garth J Thompson, Hillary Schwarb, Eric Schumacher, Regina Schmidt, Charles Epstein, Shella D Keilholz

**Affiliations:** 1 Biomedical Engineering, Emory University & Georgia Institute of Technology, Atlanta, GA 30306, USA; 2 School of Psychology, Georgia Institute of Technology, Atlanta, GA 30332, USA; 3 Air Force Research Laboratory, Wright-Patterson Air Force Base, OH 45433, USA; 4 Neurology, Emory University Hospital, Atlanta, GA 30322, USA

## 

**Figure 1:**
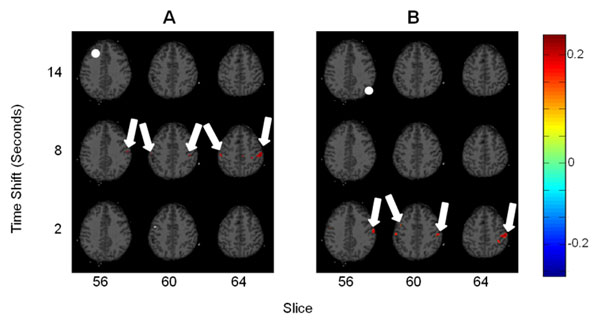
fMRI to EEG correlation for two electrodes. Slice number and time shift are shown on the abscissa and ordinate, respectively. Approximate electrode location is indicated by a white circle in the upper left slice. Arrows point to areas of significant correlation. **A:** Correlation between the AF3 electrode and BOLD signal. **B:** Correlation between the PO8 electrode and the bold signal.

Recently, researchers have taken interest in simultaneously recording electroencephalography (EEG) and functional magnetic resonance imaging (fMRI) to explore the relationship between the blood oxygen level dependent (BOLD) signal and underlying neuronal activity[1]. Many studies investigate BOLD signal relationship to high-pass (> 1 Hz) filtered EEG data, but little work on has been done on slow (< 1Hz) cortical potential correlates of fMRI because low frequency EEG data are commonly discarded as drift artifacts[1]. Nevertheless, much slower (< 0.1 Hz) components of the fMRI signal are used to establish functional resting state networks (RSNs) within the brain[2]. In this study, five subjects underwent simultaneous recording of EEG and fMRI in a resting state, lying quietly with eyes open. Two ten minute scans were acquired per subject over the entire brain (TR=2 seconds, TE=30 milliseconds, 64x64 voxels, 33 slices). After standard functional connectivity preprocessing[3], the BOLD signals were band-pass filtered between 0.01 – 0.08 Hz. EEG data were obtained from a 64-channel electrode montage in a standard International 10-10 System configuration. Each signal was then filtered between 0.01 – 0.08 Hz and de-noised of scanner and ballistocardiographic artifacts. Channels were clustered using k-means and each cluster’s signal was calculated in order to be regressed from its respective electrodes. All EEG data were resampled to 0.5 Hz for comparison with functional data. Pearson correlation was then computed between pairs of individual EEG electrodes. Two electrode channels (AF3 and PO8) were chosen to be cross-correlated with BOLD signals at various time shifts between -10 – 20 seconds, due to their consistent presence in anticorrelated clusters from the paired EEG clustering. This was performed both with and without EEG cluster signal removal, the former allowing better visualization of correlation results. Finally, all subjects’ electrode-specific EEG-fMRI correlations were averaged together and two maps of correlation were produced (Figure 1). These maps were corrected for multiple comparisons using a false discovery rate of 0.05, assuming a normal distribution of correlation values. The relationship between BOLD signals and slow cortical potentials can be observed over the sensorimotor cortex for both AF3 and PO8, demonstrating a correlation between EEG and a known RSN[2]. The time delays in correlation may also be related to previously observed BOLD signal propagations[3].
